# Inferring Condition-Specific Modulation of Transcription Factor Activity in Yeast through Regulon-Based Analysis of Genomewide Expression

**DOI:** 10.1371/journal.pone.0003112

**Published:** 2008-09-03

**Authors:** André Boorsma, Xiang-Jun Lu, Anna Zakrzewska, Frans M. Klis, Harmen J. Bussemaker

**Affiliations:** 1 Swammerdam Institute for Life Sciences, University of Amsterdam, BioCentrum Amsterdam, Amsterdam, The Netherlands; 2 Department of Biological Sciences, Columbia University, New York, New York, United States of America; 3 Center for Computational Biology and Bioinformatics, Columbia University, New York, New York, United States of America; Genome Institute of Singapore, Singapore

## Abstract

**Background:**

A key goal of systems biology is to understand how genomewide mRNA expression levels are controlled by transcription factors (TFs) in a condition-specific fashion. TF activity is frequently modulated at the post-translational level through ligand binding, covalent modification, or changes in sub-cellular localization. In this paper, we demonstrate how prior information about regulatory network connectivity can be exploited to infer condition-specific TF activity as a hidden variable from the genomewide mRNA expression pattern in the yeast *Saccharomyces cerevisiae*.

**Methodology/Principal Findings:**

We first validate experimentally that by scoring differential expression at the level of gene sets or “regulons” comprised of the putative targets of a TF, we can accurately predict modulation of TF activity at the post-translational level. Next, we create an interactive database of inferred activities for a large number of TFs across a large number of experimental conditions in *S. cerevisiae*. This allows us to perform TF-centric analysis of the yeast regulatory network.

**Conclusions/Significance:**

We analyze the degree to which the mRNA expression level of each TF is predictive of its regulatory activity. We also organize TFs into “co-modulation networks” based on their inferred activity profile across conditions, and find that this reveals functional and mechanistic relationships. Finally, we present evidence that the PAC and rRPE motifs antagonize TBP-dependent regulation, and function as core promoter elements governed by the transcription regulator NC2. Regulon-based monitoring of TF activity modulation is a powerful tool for analyzing regulatory network function that should be applicable in other organisms. Tools and results are available online at http://bussemakerlab.org/RegulonProfiler/.

## Introduction

About a decade ago, simultaneous measurement of the transcript level of all genes in a genome using DNA microarrays became technically feasible [1], [2]. Since then, a large amount of data from such experiments has been accumulated in public repositories [3], [4]. More recently, the marriage between chromatin-immunoprecipitation and microarray technology (“ChIP-chip”) [5], [6] has made it feasible to measure the genomewide profile of *in vivo* binding by transcription factors (TFs) [7], [8]. Methods for measuring *in vitro* TF-DNA binding affinities have also been developed [9]–[11]. Finally, a number of large-scale TF deletion and over-expression studies have been performed [12]–[14]. Consequently, genomewide information about the connectivity between TFs and their target genes is increasingly available.

The rate at which a gene is transcribed is controlled by transcription factors (TFs) binding to its upstream promoter region. Knowledge about how TF activity is modulated in a condition-specific manner by signaling pathways is therefore crucial for understanding gene regulatory network function. It is widely recognized that TF activity is often regulated at the post-translational level. First, the regulation of translation or of protein turnover rate may cause the protein abundance to not be proportional to mRNA abundance. Experimental quantification of protein abundance may depend on antibody availability and is not easily done on a high-throughput scale. Second, ligand binding or non-covalent modification and subsequent translocation between nucleus and cytoplasm can affect TF activity even at constant total cellular protein abundance. For all these reasons it is challenging to measure TF activity directly. Network inference algorithms therefore often use the mRNA expression level of the gene that encodes a TF as a proxy for that TF's regulatory activity [15], [16].

If prior knowledge about which genes are the targets of a specific TF is available, an alternative and potentially more accurate approach can be taken. As several studies have shown, it is possible to infer modulation of the “hidden” activity of a TF from the genomewide changes in mRNA expression, using either motif analysis of upstream promoter sequences [17], [18] or ChIP-chip data [19], [20] to estimate the connectivity between a TF and its target genes (for a recent review, see [21]).

We previously developed a simple web-based tool named *T-profiler* that scores differential expression of predefined gene sets using the two-sample t-test [22], [23]. Conceptually similar to Gene Set Enrichment Analysis [24], *T-profiler* was originally developed for scoring differential expression of Gene Ontology categories [25]. However, it can also infer condition-specific modulation of post-translational TF activity when used in conjunction with gene sets consisting of putative TF targets. These “regulons” can be defined either based on upstream matches to a consensus binding motif or based on the results of a ChIP-chip experiment.

In this paper, we perform a detailed assessment of the biological utility of our regulon-based approach. We first validate experimentally that *RegulonProfiler* can detect modulation of TF activity. Next, we create a database containing t-values that quantify the differential expression of a large number of regulons across a compendium of expression data for the yeast *S. cerevisiae*. Querying this database allows us to determine which TFs are modulated in a given experiment, or conversely, by which environmental conditions a given TF is modulated. We quantify the degree to which the mRNA expression level of each TF is predictive of its regulatory activity, and find a wide range of behaviors. We also organize TFs into “co-modulation networks” based on their inferred activity profile across conditions, and find that this reveals functional and mechanistic relationships. Finally, we present evidence that the PAC and rRPE motifs antagonize TBP-dependent regulation, and function as core promoter elements governed by the transcription regulator NC2. Taken together, these results demonstrate the value of regulon-based, TF-centric, analysis of the yeast regulatory network.

## Results

### Creating a database of inferred TF activities

**Figure 1:**
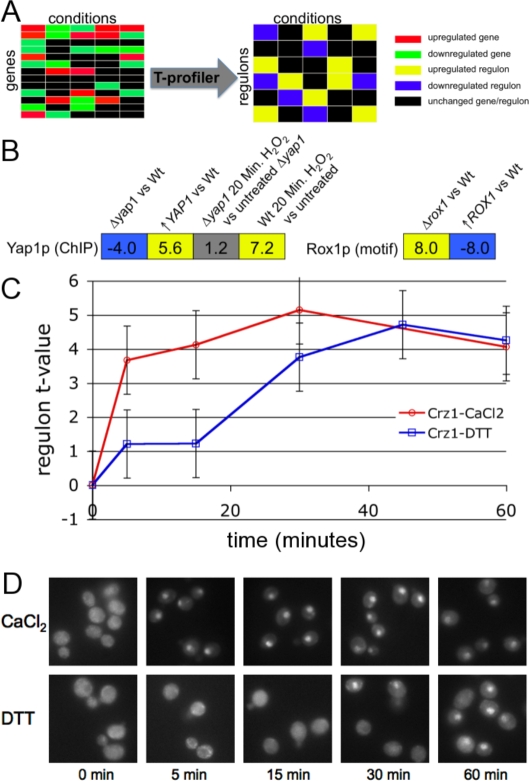
Validation of inferred TF activity modulation. (A) Schematic diagram showing how *T-profiler* [22] was used to convert each genomewide mRNA expression profile to a set of t-values that quantify the change in regulatory activity for each TF for which a set of putative targets (“regulon”) was available. The results are available at http://bussemakerlab.org/RegulonProfiler/. (B) Change in regulatory activity of the activator Yap1p and the repressor Rox1p when the corresponding factors are deleted or overexpressed, as inferred by *T-profiler*. The *t*-values for Yap1p are based on the ChIP-based regulon (rich medium), while for Rox1p a motif-based (YCTATTGTT) regulon was used. As expected, a Yap1p regulon response is observed for wild-type cells stressed using H_2_O_2_, but not for Δ*yap1* cells in the same condition. (C) Timing of the activation of the Crz1p motif-based gene set during CaCl_2_ [31] and DTT [27] stress. Note that the t-value for each time point is derived from a distinct genomewide expression profile. (D) Cellular localization of Crz1p during CaCl_2_ (upper panel) and DTT stress (lower panel) assayed using fluorescence microscopy. We used DAPI staining (data not shown) to confirm that the small bright spots to which GFP-tagged Crz1p has translocated are the nuclei of the cells.

We used *T-profiler* [22] to populate a database of t-values that quantify the change in mean expression for a large number of predefined gene sets across a large number of experimental conditions (**Figure 1A**). For genes sets, we used both “motif-based” regulons, defined based on matches to specific consensus motifs in their 600-base pair upstream regions, and “ChIP-based” regulons, defined based on measurements of promoter occupancy in different conditions by Harbison *et al.* [7]. We analyzed a wide variety of experiments, including cell cycle [26], various stress response time courses [27], and a collection of gene deletion and gene suppression experiments [28], [29]; see Materials and Methods and Supplementary Figure S1 for details. The full results of our analysis are available at http://bussemakerlab.org/RegulonProfiler/.

### Validation of inferred condition-specific TF activity modulation

We first tested the ability of *T-profiler* to infer changes in TF activity by analyzing experiments in which a transcription factor-encoding gene was either deleted or over-expressed. Yap1p activates genes involved in the response to oxidative stress, while Rox1p represses genes upon oxygen limitation. We monitored the t-values of the ChIP-based Yap1p (YPD condition) regulon (72 genes) and the motif-based (YCTATTGTT) Rox1p regulon (95 genes); see **Figure 1B**. In a *YAP1* deletion strain, significant down-regulation (*t*-value = −4.0; *E*-value = 0.015) of the Yap1 regulon is observed, while over-expression of *YAP1* results in its upregulation (*t*-value = 5.6; *E*-value = 6*10^−6^). Conversely, deletion of the repressor gene *ROX1* results in upregulation of the Rox1p regulon, while overexpression of *ROX1* causes downregulation. The specificity of our method is demonstrated by the lack of a Yap1p regulon response in H_2_O_2_-stressed Δ*yap1* cells.

We also tested *T-profiler* predictions concerning the time-dependent modulation of Crz1p, which is known to translocate to the nucleus in response to activation by calcineurin [30]. **Figure 1C** shows the activity of the motif-based (GAGGCT) Crz1p regulon in response to CaCl_2_ [31] and dithiothreitol (DTT) [27], respectively. Upon both CaCl_2_- and DTT-induced stress, Crz1p is activated, but with CaCl_2_ an immediate response (within 5 minutes) is seen, while with DTT the response is considerably delayed. To validate these predictions, we used a GFP-tagged Crz1 protein and fluorescence microscopy (see Materials & Methods). In both cases, we were able to confirm the timing of the measured responses (**Figure 1D**).

### Condition-specific modulation of Hac1p regulatory activity

**Figure 2:**
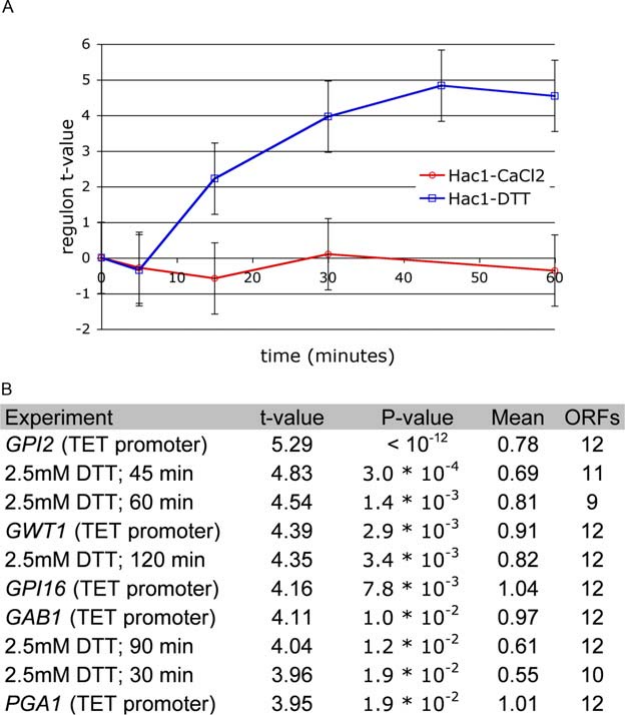
Condition-specific activity of the Hac1p regulon. (A) Transcriptional response of the Hac1p regulon (ChIP-based) during DTT [27] and CaCl_2_ [31] stress. (B) The top ten expression profiles (out of 936), ranked by the t-value for the Hac1p regulon. These expression profiles are either from DTT-stressed cells [31] or from cells with a partially suppressed essential gene under control by the TET-promoter [29].

Our database can be used to perform queries that reveal condition-specific activation of specific TFs. We illustrate this for the Hac1 regulon. Cells treated with DTT have to cope with reductive stress resulting in accumulation of misfolded proteins in the endoplasmic reticulum [32]. This leads to the activation of the unfolded protein response, which is governed by the transcription factor Hac1p [33]. **Figure 2A** shows the temporal profile of activation of the ChIP-based Hac1 regulon under DTT stress [27]. This response is independent of the aforementioned Crz1p response and therefore does not occur during CaCl_2_ stress. Next, by ranking all experiments according to the t-value of the Hac1p regulon, we found that the Hac1p is specifically activated in DTT-stressed cells or in cells in which specific essential genes have been partially suppressed [29] (**Figure 2B**). *GPI2* and *GWT2* function in GPI-anchor biosynthesis, whereas *GPI16* and *GAB1* are involved in transferring pre-assembled GPI-anchors to a specific class of secretory proteins called GPI-proteins; when these processes do not function properly, defective GPI-proteins accumulate in the endoplasmatic reticulum (ER). *PGA1* codes for a protein that localizes to the nuclear periphery, a subregion of the ER; when its activity is repressed, maturation of the GPI-protein Gas1p and of Pho8p, which also follows the secretory pathway, is affected [34], likely resulting in their accumulation in the ER. In other words, activation of the Hac1p regulon seems to occur specifically when defective proteins accumulate in the ER. The condition-specific activation of the Hac1p regulon is just one of many discoveries that can be made about the transcription network by exploring our database of inferred TF activities.

### Relationship between mRNA expression level and regulatory activity of a TF

**Figure 3:**
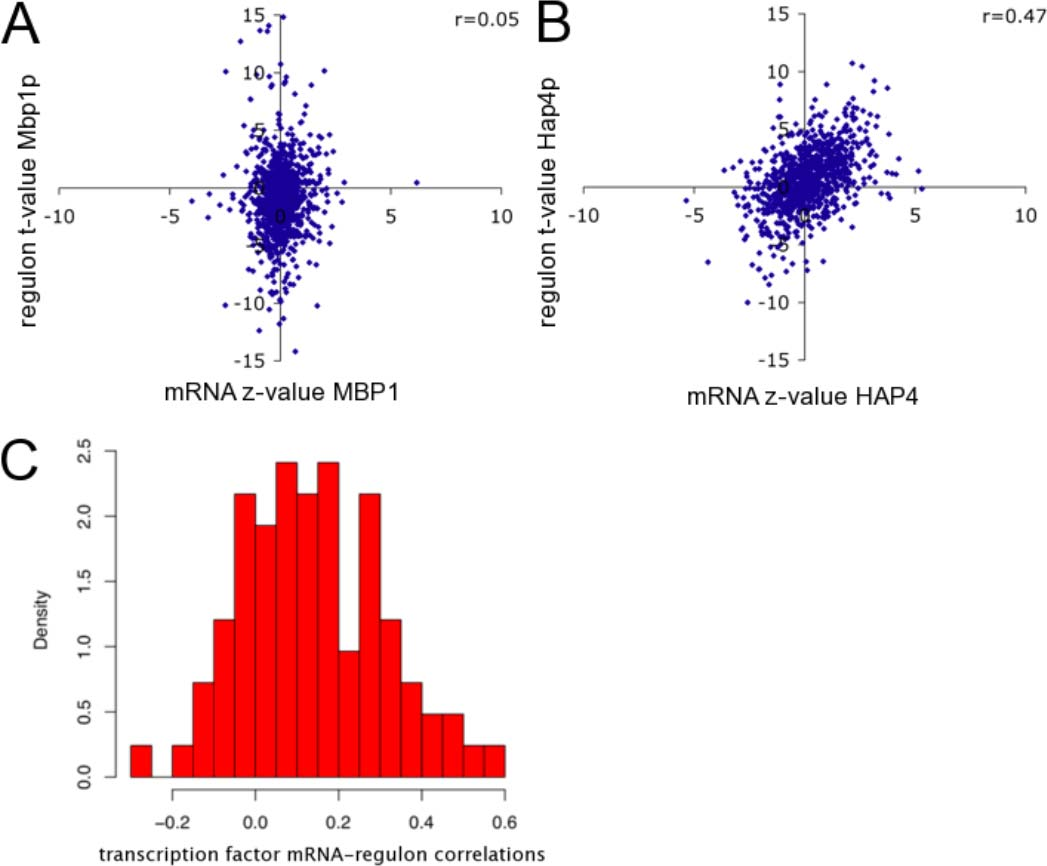
Relationship between mRNA expression level and regulatory activity of a TF. Shown are Pearson correlations between the normalized mRNA expression log-ratio and inferred activity (ChIP-based regulon) across all 936 expression profiles of (A) *MBP1* (marginal correlation: *r* = 0.05; *P* = 0.14) and (B) *HAP4* (strong correlation: *r* = 0.47; *P*<10^−12^). (B) Distribution of the correlations shown in part (a) and (b) over all 83 TFs analyzed.

Having established that regulon-based analysis using *T-profiler* allows us to quantify the post-translational regulatory activity of TFs, we explicitly addressed the question to what extent mRNA expression level can be used as a proxy for activity. The results shown in Figure 1d indicate that the activation of Crz1p is regulated by translocation to the nucleus. Indeed, only a marginal correlation (*r* = 0.08; *P* = 0.015) exists between the mRNA expression and inferred activity of Crz1p over all conditions in our database. Using ChIP-based regulons, we were able to quantify the degree to which mRNA expression level is predictive of post-translational activity for 83 distinct TFs. **Figure 3A** shows an example where the mRNA level is a poor predictor of TF activity (Mbp1; *r = *0.05, *P* = 0.14). By contrast, **Figure 3B** shows that the mRNA levels of Hap4 are a good predictor for its inferred activity (*r = *0.47; *P*<10^−12^). In **Figure 3C** the distribution of mRNA level vs. regulon activity correlations across all TFs is shown, revealing that whether or not mRNA expression is a valid proxy for activity strongly depends on the identity of the TF (see **Supplementary** **Table S1** for full results).

### Organizing TFs into “co-modulation networks” based on their activity profile

**Figure 4:**
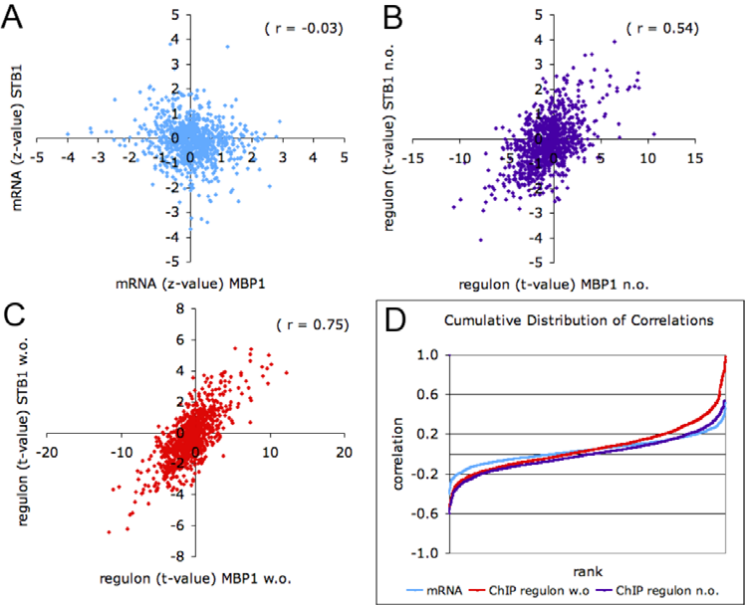
Co-modulation of transcription factors. (A) There is no significant correlation between the normalized mRNA log-ratios (z-scores) over all experiments of the transcription factor genes *STB1* and *MBP1* (*r* = −0.03; *P* = 0.36). (B) By contrast, the inferred activities of the ChIP-based regulons (t-values) of Stb1p and Mbp1p over all experiments are highly correlated (*r* = 0.53; *P*<10^−12^) when overlapping genes are removed (“n.o.” indicates no overlap). (C) As expected, the correlation is even stronger (*r* = 0.75; *P*<10^−12^) when overlapping genes are included (“w.o.” indicates with overlap). (D) Cumulative distribution of pairwise correlations across all transcription factor pairs, for each of the co-modulation detection metrics used in parts A–C, as indicated by the color of the graph.

For each TF, the inferred activity profile over roughly a thousand conditions represents a highly specific regulatory signature. It is highly unlikely for two such activity profiles to be similar, unless (i) they are derived from strongly overlapping regulons, or (ii) the corresponding TFs are modulated by the same signaling pathway. The latter case suggests a way of organizing the TFs into a network based on co-modulation of their post-translational activity. To illustrate this, consider the cell cycle regulators Stb1p and Mbp1p. The correlation between their mRNA expression values (*r* = −0.03; *P* = 0.36) (**Figure 4A**) over all conditions in our database is not statistically significant. However, the t-values scoring the differential expression of the ChIP-based regulons for Mbp1p (188 genes) and Stb1p (63 genes) are highly correlated (*r = *0.75; *P*<10^−12^) (**Figure 4C**). Even when we exclude the 23 genes that occur in both regulons, the correlation remains high (*r* = 0.54; *P*<10^−12^) (**Figure 4B**).

The cumulative distributions in Figure 4d show how the three methods of quantifying TF co-modulation compare across all pairs of TFs (see **Supplementary** **Table S2** for full results). As expected, the regulons with overlapping genes included show the strongest correlation, but only on the positive end of the distribution. Despite the very strict treatment of removing all overlapping genes, the correlation of regulons with overlapping genes removed is slightly better than the mRNA-based correlation at the positive end of the distribution, and are dramatically better at the negative end. Taken together, these results indicate that implicit information about the connectivity between signal transduction pathways and transcription factors can be obtained by comparing the activity profiles of TFs.

**Figure 5:**
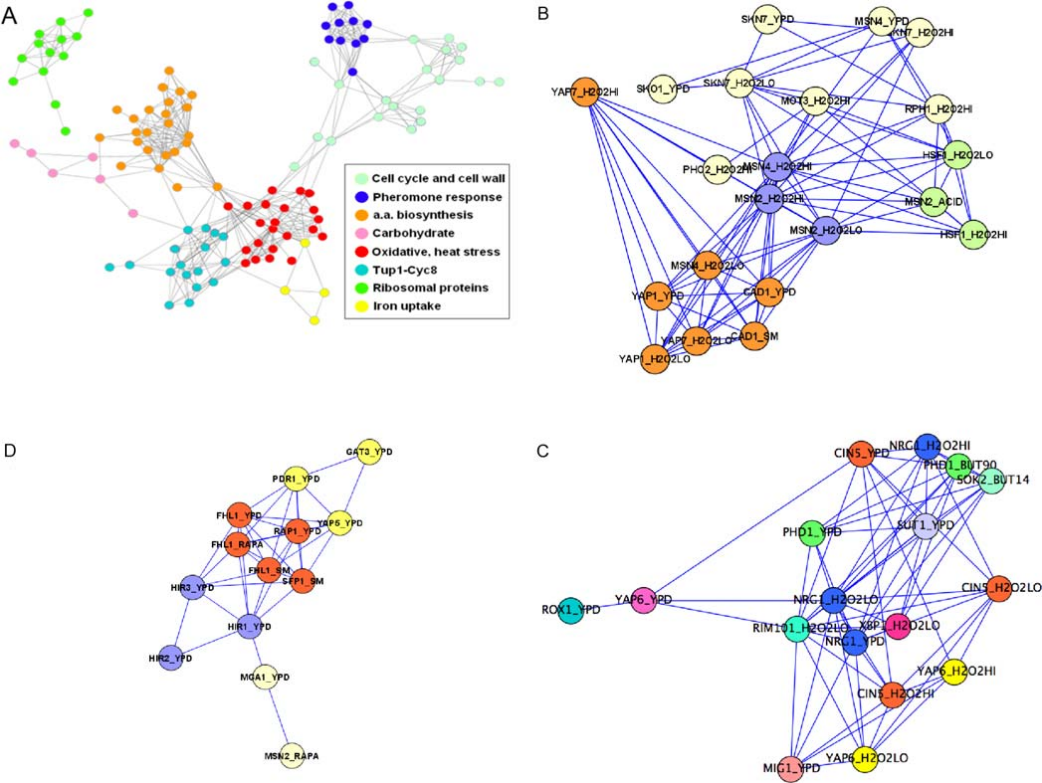
Co-modulation networks derived from inferred TF activity profiles. (A) The network obtained by connecting all ChIP-based regulons whose t-profiles across the 936 conditions in our database are strongly correlated (*r*>0.5). To visualize the network, we applied the yFiles organic layout setting of Cytoscape [56]. Colors represent functionally related transcription factors (see legend). Eight separate sub-networks can be distinguished. (B) The oxidative and heat stress sub-network. In the label of each node, the condition used in the ChIP-chip experiment on which each regulon is based [7], is indicated in addition to the name of the TF. The color-coding is as follows. In green: regulons that mainly contain heat stress genes; in orange: regulons that mainly contain oxidative stress genes; the Msn2/4 ChIP- based regulons (blue) interconnect both; in purple: the Skn7 regulons; the regulons shown in yellow do not have a clear functional bias. (C) The “Tup1p-Cyc8p” sub-network (lower left cluster in Figure 4), in which TFs that rely on this co-repressor to control their transcriptional targets are connected. (D) The “ribosomal protein” sub-network (upper left cluster in Figure 4). The histone-regulating factors Hir1p/Hir2p/Hir3p are connected to the ribosomal protein-regulating factors Rap1p-Fhl1p-Sfp1p. In (b) and (c), similar colors again represent similar biological function.

Starting from ChIP-based activity profiles for a large number of TFs, and drawing connections between pairs of TFs only when the correlation between their activities exceeds a stringent threshold (*r*>0.5), we organized all TFs into a “co-modulation network” consisting of eight disjoint sub-networks (**Figure 5A** **;** see **Supplementary** **Table S3** for full results in Cytoscape format). In agreement with findings by Luscombe *et al*. [35], the cell-cycle sub-network and the pheromone response sub-network are found to be separated from the other sub-networks, whereas the oxidative/heat stress sub-network takes a central position. The most highly connected transcription factors are Msn4p (with 21 interactions) and Msn2p, Gcn4p, and Skn7p (each with 20 interactions). Within the oxidative-heat stress sub-network (**Figure 5B**) there is a separation between transcription factors involved in oxidative stress (Yap1p, Yap7p and Cad1p) and heat stress (Hsf1p). This sub-network also contains Skn7p, which has been previously described as being involved in oxidative, heat and osmotic stress [36].

One of the other sub-networks in Figure 5a contains Sut1p, Nrg1p, Phd1p, Rim101p and Sok2p (**Figure 5C**). These TFs are involved in a variety of stress responses. However, a shared feature is that most of them are known to repress gene transcription by interacting with the co-repressor Tup1p-Cyc8p (Ssn6p). We analyzed the expression profiles of both the *tup1* and *cyc8* deletion mutant [28], and found that almost all of the ChIP-based regulons in this sub-network are indeed de-repressed in both the *tup1*Δ/wt and the *cyc8*Δ/wt expression profiles (**Table 1**). One of the members of the Tup1p-Cyc8p sub-network is Cin5p, a poorly characterized basic leucine zipper transcription factor of the yAP-1 family, which mediates pleiotropic drug resistance [37]. It is constitutively located in the nucleus. The Cin5p regulon is de-repressed in a *cin5* deletion mutant [28] included in our database. We therefore predict that Cin5p interacts with the Tup1p-Cyc8p co-repressor complex to negatively regulate its target genes.

**Table 1:** Regulon analysis of the *tup1*Δ and *cyc8* (*ssn6)*Δ transcription profiles.

*tup1*Δ/wt		*cyc8*Δ/wt	
TF (condition)	t-value	TF (condition)	t-value
NRG1 (YPD)*	**14.8**	SOK2 (BUT 14)*	**9.6**
RIM101 (H2O2 low)*	**14.5**	NRG1 (YPD)*	**9.6**
CIN5 (H2O2 low)*	**13.9**	YAP6 (YPD)*	**8.6**
NRG1 (H2O2 low)*	**13.6**	NRG1 (H2O2 low)*	**8.6**
YAP6 (H2O2 low)*	**12.2**	PHD1 (BUT 90)*	**8.5**
SOK2 (BUT 14)*	**11.6**	CIN5 (H2O2 low)*	**8.4**
YAP6 (YPD)*	**11.0**	RIM101 (H2O2 Low)*	**8.1**
PHD1 (BUT 90)*	**10.6**	NRG1 (H2O2 high)*	**8.1**
MIG1 (YPD)*	**10.6**	CIN5 (YPD)*	**8.0**
PHD1 (YPD)*	**10.6**	YAP6 (H2O2 low)*	**7.9**
NRG1 (H2O2 high)*	**9.7**	SUT1 (YPD)*	**7.5**
SUT1 (YPD)*	**9.6**	PHD1 (YPD)*	**7.5**
CIN5 (H2O2 high)*	**9.3**	CIN5 (H2O2 high)*	**6.8**
YAP6 (H2O2 high)*	**8.6**	MIG1 (YPD)*	**6.7**
CIN5 (YPD)*	**8.5**	**AFT2 (H_2_O_2_ low)**	**6.5**
YJL206C (H_2_O_2_ low)	7.5	**SKN7 (H_2_O_2_ low)**	**6.4**
**SKN7 (H_2_O_2_ low)**	**7.2**	**XBP1 (H_2_O_2_ low)**	**5.6**
**AFT2 (H_2_O_2_ low)**	**7.0**	**SKN7 (H_2_O_2_ high)**	**5.5**
**XBP1 (H_2_O_2_ low)**	**6.5**	YAP6 (H_2_O_2_ high)*	**5.4**
CUP9 (YPD)	5.9	**SKN7 (YPD)**	**5.3**
**SKN7 (YPD)**	**5.7**	RCS1 (H_2_O_2_ high)	4.6
SKO1 (YPD)	5.7	PUT3 (H_2_O_2_ low)	4.5
**SKN7 (H_2_O_2_ high)**	**5.6**	ROX1 (YPD)*	**3.9**
**YJL206C (YPD)**	**5.6**	**YJL206C (YPD)**	**3.8**
ROX1 (YPD)*	**4.8**		
YAP1 (H_2_O_2_ low)	4.1		

Shown are ChIP-based regulons with a significant t-score (*E*-value<0.05) for *tup1*Δ and *cyc8* (*ssn6*)Δ mutant vs. wild-type expression data [28]. Regulons scoring significantly in both mutants are shown in bold. The transcription factors that are part of the Tup1-Cyc8 co-modulation sub-network (Figure 5c) are marked with an asterisk. The condition of the ChIP-chip experiment [7] is shown in parentheses.

The sub-network shown in **Figure 5D** reveals the co-modulation of Rap1p, Sfp1p and Fhl1p, known to control the expression of ribosomal protein genes, and Hir1p, Hir2p, and Hir3p, which are co-repressors involved in the cell-cycle-regulated transcription of histone genes. While ribosome biogenesis has been linked to cell division via Sfp1p [38], the parallel activation of the Hir regulon detected by our co-modulation approach provides additional clues about the coupling between these two processes.

### PAC and rRPE may serve as NC2-dependent core promoter elements

**Figure 6:**
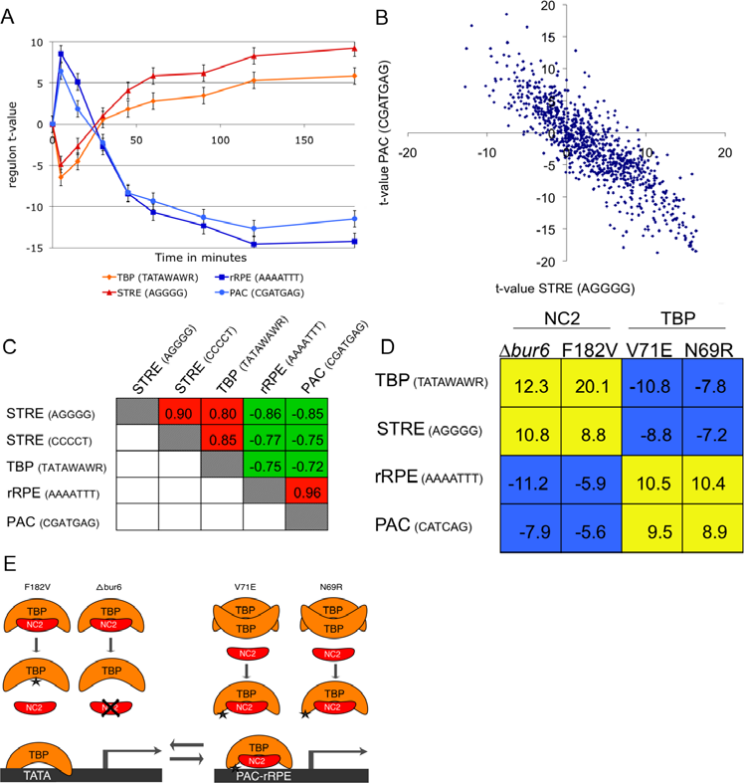
Motif-based dissection of the Environmental Stress Response. (A) Transcriptional response during DTT stress [27] of four regulons based on motifs (see text) associated with the Environmental Stress Response (ESR). (B) Scatter plot of the t-values for the 936 experiments in our database for the STRE versus the PAC regulon, showing a strong negative correlation (*r* = −0.85). (C) Strongly coupled antagonism between STRE/TBP and PAC/rRPE motifs. Shown are Pearson correlation coefficients for all pairwise comparisons of the t-value profiles for the four motifs. All *r*-values correspond to an E-value<10^−14^. (D) Evidence for interaction between PAC/rRPE and the factor NC2. Shown is the response of the four motif-based regulons to deletion of one of the components of NC2 (*bur6*Δ), as well as mutations to TBP that affect its dimerization (V71E and N69R) or its interaction with NC2 (F182V). (E) Hypothetical binding of TBP/NC2 to the TATA and PAC-rRPE (core) promoter. The left panel illustrates the inability of the TBP mutant F182V and the NC2 mutant Δbur6 to form a TBP/NC2 complex, as a consequence of which more TBP is available to bind and initiate transcription at TATA-containing promoters. The right panel illustrates the inability of the TBP mutants V71E and N69R to form the inactive homodimeric TBP complex, causing more TBP/NC2 complexes to be formed, which (directly or indirectly) stimulates transcription from PAC/rRPE promoters.

Besides the specific response of the Hac1p gene set to DTT stress, a general transcriptional program known as the Environmental Stress Response (ESR) is triggered [27]. Motifs associated with the ESR include the stress-response element (STRE) motif (AGGGG/CCCCT) bound by the transcription factor Msn2p [39], PAC (CGATGAG) [40], and rRPE (AAAATTT), which is associated with genes required for rapid growth [41]. **Figure 6A** shows activity profiles for the corresponding gene sets during DTT stress. Further analysis of the activity profiles of the ESR motifs reveals that the antagonism between STRE and PAC/rRPE observed during DTT stress holds over a wide range of cellular states (**Figure 6B,C**). The TATA-box gene set (TATAWAWR) correlates strongly positively with STRE (r = 0.80), consistent with recent observations by Basehoar et al. [**42**] that TATA-box containing genes are activated in response to various stresses.

The strongly coupled, but opposing transcriptional behavior of the STRE/TBP and PAC/rRPE gene sets across many conditions suggests a mechanistic relationship. Currently, it is not known which gene specific transcription factors bind to the PAC element. Although Stb3p has been found to bind the rRPE element, this only applies for a small portion of the rRPE containing genes [43]. Similar to the TBP motif, the PAC and rRPE elements are predominantly found in the first 150 bp upstream from the translational start site [44]. Promoter regions of genes containing PAC and rRPE elements are generally TATA box-less. Beer and Tavazoie [44] found that PAC and rRPE elements correlate with expression only when the PAC element is located downstream of the rRPE element. Similar motif characteristics have been described for regulatory sequences in *Drosophila* named DPE (Downstream core Promoter Element), which serve as core promoter elements [45]. The DPE is bound by NC2, a bi-functional general transcription factor that differentially regulates gene transcription through DPE or TATA-box motifs [46]. NC2 is a heterodimer of two histone-fold subunits. In *S. cerevisiae*, the α-NC2 subunit consists of Bur6p and Ydr1p, while the β-NC2 subunit consist of Ncb2p. **Figure 6D** shows that expression profiles of bur6Δ [47] cells show strong induction of the TBP (TATAWAWR) (t-value = 12.3) and STRE (AGGGG) gene sets (t-value = 10.8) and strong repression of the PAC (CGATGAG) and rRPE (AAAATTT) gene sets (t-values = −7.9 and −11.2, respectively). The expression profile of a TBP mutant (F182V; [48]) that is unable to bind NC2 shows similar behavior. The opposite pattern is observed for TBP mutants V71E and N69R, which are unable to dimerize. Since TBP dimers are inactive, this will increase the amount of NC2-TBP complex, which in turn represses transcription of TATA-box regulated genes and induces transcription via the PAC and rRPE element (**Figure 6E**). Together, these observations suggest that the PAC and rRPE sequences may function as core promoter elements with similar properties as DPE, and that in *S. cerevisiae*, NC2 may play a similar role as in *Drosophila*, where it activates DPE-driven promoters and represses TATA-box driven promoters [46].

## Discussion

In this study we scored differential expression at the level of gene sets to infer changes in the activity of transcription factors from the mRNA expression levels of the genes predicted to be under their control, based either on upstream sequence matches to cis-regulatory elements (motif-based regulons) or on occupancy by a specific transcription factor (ChIP-based regulons). We created a database of inferred regulatory activities for a large number of TFs under a wide variety of stress conditions and gene deletion mutants in budding yeast, and used it to perform TF-centric analysis of the yeast regulatory network.

Whether the ChIP-based or motif-based regulon performs better depends on the identity of the TF and possible also the expression profile analyzed. It is difficult to make a general statement. However, the t-values reported by our website make it easy for the user to compare the performance for any TF/experiment combination of interest.

We have validated our computational approach both computationally and experimentally. First, we confirmed that deletion and over-expression of two transcription factors (an activator and a repressor) resulted in the expected up- and down-regulation of their accompanying gene groups. Second, using fluorescence microscopy we were able to observe the translocation of two transcription factors to the nucleus during calcium and DTT treatment, in agreement with the *T-profiler* predictions.

DTT stress also activates a specific response of a gene group regulated by the Hac1p transcription factor, a response that does not occur in cells treated with calcium. In fact, querying our database for experiments, in which the Hac1p-based gene group is activated, only revealed 11 experiments with significant t-values. Four of those originate from the DTT time course, while the others are from transcription profiles of partially suppressed essential genes. Interestingly, these genes are either involved in GPI-anchor biosynthesis, GPI-anchor addition, or in GPI-protein maturation. Another example is that the Rlm1p-based gene group is mainly activated in experiments related to cell wall perturbation, caused by, for example, Calcofluor white or Zymolysase [49], or in deletion mutants defective in cell wall formation [50]. Such use of our database to query for condition specific activation bears some resemblance to the “connectivity map” approach [51], which related a compendium of drug related gene expression signatures (represented as gene sets) to the expression profiles of gene deletions and disease.

To further analyze functional relationships between TFs, we used inferred activity TF profiles across a large number of conditions to organize TFs into a “co-modulation network” consisting of a number of disjoint sub-networks. In agreement with the results of Luscombe et al. [35] we found the cell-cycle and pheromone sub-network to be separated from the other sub-networks. The advantage of inferring TF activities as hidden variables was illustrated for the transcription factors Mbp1p and Stb1p, which show poor correlation at the mRNA level but strong correlation at the regulon activity level. Recognizing that such correlation might be caused by overlap between the regulons, we removed the 23 genes that occurred in both regulons and recomputed the correlation, which remained high. Tomlins *et al*. [52] were able to use a method purely based on the overlap between gene groups from various sources to build an interaction network that yielded new insights on prostate cancer progression. This suggests that while our co-modulation network approach provides useful biological information about TF-TF associations even if there is no overlap between regulons, the contribution to the regulon-regulon correlation from the overlapping genes is also biologically meaningful.

In contrast to the condition-specific activity of many regulons, those based on the STRE motifs (AGGGG/CCCCT) and TBP (TATAWAWR) are activated in 50% of all conditions and are therefore regulated in a more general manner. Compared to the STRE and TBP-regulons, the PAC and rRPE regulons show opposite transcriptional behavior. The observed bipolar transcriptional regulation in *Saccharomyces cerevisiae* is also found by others [42].We propose that there is a mechanistic relationship between the regulation of these motif gene groups and provide evidence that NC2, a bi-functional transcriptional regulator that binds TBP, could serve as the mechanistic link. Basehoar *et al*. [42] showed that approximately 20% of yeast genes contain a TATA box, and similar numbers have also been found for higher eukaryotes [53]. It might be interesting to determine to what extent this form of regulation is conserved in higher eukaryotes.

While the results reported here are limited to the yeast *S. cerevisiae*, we expect our approach to be valid in other organisms as well, including human. Whenever prior information about which genes are directly targeted by a TF is available, regulon-based analysis of differential expression using T-profiler should allow the “hidden variables” that represent the true post-translational activity of the TF to be estimated from the genomewide expression profile.

## Materials and Methods

### Definition of gene sets

We performed *T-profiler* analysis as described in [22] using motif and ChIP-chip based regulons. Motif-based regulons were defined as sets of genes with a match to a particular consensus motif within the 5′ 600 base pairs upstream of the ORF [54], allowing no overlap between neighboring ORFs. The consensus motifs used in T-profiler [22] are derived from three different sources. First, motifs were extracted from the SCPD database (http://rulai.cshl.edu/SCPD/). Next, motifs were found by comparing the genome sequence of highly related yeast species [27], [55]. Finally, motifs discovered in various microarray experiments by the REDUCE algorithm [17] were added. Most of these motifs are similar or identical to motifs described in the literature. In total, 115 motif sets have been included in T-profiler calculations. To define the ChIP-based regulons, we used the transcription factor binding data obtained by Harbison *et al*. [7]. This data set contains ChIP-chip results of 203 transcription factors from experiments performed in rich medium (YPD). In addition, 84 of these transcription factors were also assayed in one or more of 12 other environmental conditions; therefore, multiple ChIP-chip regulons may be defined for the same TF. A gene was considered to be part of the regulon if the p-value reported by the authors was smaller than 0.001. ChIP-based regulons were required to have at least 7 members, yielding a total of 252 gene sets that were used for T-profiler analysis.

### Expression library of transcription profiles

Our compendium of *S. cerevisiae* expression profiles contains data for 936 cellular conditions from 19 publications, obtained using different microarray platforms such as Genefilter, Affymetrix, and spotted slides. Details can be found at (http://bussemakerlab.org/RegulonProfiler/).

### Co-modulation network

To quantify the similarity of pairs of inferred TF activity, we computed the Pearson correlation *r* between the t-values for the corresponding regulons across all conditions in our expression library. For each value of *r*, the test statistic
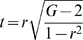
was computed, and a two-tailed *P*-value was determined by using the t-distribution with *G*-2 degrees of freedom, where *G* is the number of genes. We only considered regulons that had significant t-values (*P*<0.05) in at least 5 experiments. We used the yFiles organic layout setting of Cytoscape [56] to create and visualize the co-modulation network.

### Fluorescence microscopy

#### Strains

GFP-fused strains, YNL027W (GFP-Crz1p) and YMR037C (GFP-Msn2p) were from Invitrogen. Strain background: EY0986 ATCC 201388: ***MAT*** **a** *his3*Δ1 *leu2*Δ0 *met15*Δ0 *ura3*Δ0 (S288C).

#### Medium and growth conditions

YPD (1% yeast extract, 2% Bactopeptone, 2% glucose) was used. YPD containing either 0.4 M CaCl_2_ or 5 mM dithiothreitol (DTT; Boehringer, Manheim) was mixed with an equal volume of YPD to achieve a final concentration of 0.2 M CaCl_2_ or 2.5 mM DTT. YPD containing 0.4 M CaCl_2_ was buffered to pH 5.0 with 7.5 mM succinate to prevent precipitation of CaPO_4_. Cultures were grown at 30°C and shaken at 250–300 rpm. The culture volume did not exceed 25% of the flask capacity. Cultures were grown to an OD of 0.5 before mixing with equal volumes of either CaCl_2_ or DTT. For CaCl_2-_treated cells, samples were taken at 0, 5, 15, 30, and 60 minutes, and for DTT-treated cells, samples were taken at 0, 5, 15, 30, 45, 60, 90, 120, and 180 minutes. For both stress conditions, the experiments from the original papers were repeated (CaCl_2_ [31], DTT [27]).

#### Cell Fixation and Microscopy

875 µl of culture were combined with 16% EM grade paraformaldehyde to a final concentration of 2% w/v and mixed for 15 minutes at 25°C. The cells were spun down for 2 minutes. The cell pellet was resuspended and washed in 1 ml of a 0.1 M KP_i_ (pH = 7.5)/1 M sorbitol buffer. Finally, the pellet was resuspended in 50 µl of this buffer and stored at 4°C until use.

Three µl of cell suspension were mounted on a glass slide under a coverslip. Microscopic imaging was performed using a CoolSnap fx cooled CCD camera, mounted on an Olympus BX60 fluorescence microscope (Olympus, Tokyo, Japan) using a phase-contrast 100× oil-immersion objective with NA = 1.3 (UPlan Fl). Fluorescence was excited with a 100 W mercury lamp; for GFP-pictures a U-MNB narrow-band cube (excitation 470–490 nm; emission >515 nm) was used. For DAPI-stained cells, 4′,6-diamidino-2-phenylindole dihydrochloride hydrate (DAPI) was added to a final concentration of 0.5 µg/ml. For DAPI pictures, a U-MWU wide-band cube (excitation 330–385 nm; emission >420 nm) was used.

## Acknowledgments

We would like to thank Gabor Halasz, Junbai Wang, Ron Tepper, Daniel Vis, and Gertien Smits for a critical reading of the manuscript, and Conrad Woldringh and Wijnand Takkenberg for their help with the fluorescence microscopy experiments.

## Supporting Information

Figure S1 Schematic overview of RegulonProfilerDB.
(0.30 MB PDF)

Table S1 Pearson correlation across all experimental conditions in RegulonProfilerDB between the t-value of the ChIP-based regulon for a particular transcription factor and the mRNA expression log-ratio of the gene encoding the same factor.
(0.00 MB TDS)

Table S2 Pearson correlation between either the mRNA expression log-ratios or the t-values of ChIP-based regulons (YPD condition only) for all pairs of transcription factors. The ranked data was used as input for Figure 4d.
(0.26 MB XLS)

Table S3 Cytoscape input file that can be used to visualize the transcription factor co-modulation network.
(0.02 MB ZIP)
