# Supplementary material for: Inferring Condition-Specific Modulation of Transcription Factor Activity in Yeast through Regulon-Based Analysis of Genomewide Expression
Source: PLoS One. 2008 Sep 3;3(9):e3112. doi: 10.1371/journal.pone.0003112 (PMC2518834; doi:10.1371/journal.pone.0003112)

Expression Data
(936 conditions)

Motif-based Regulons
Database of 124 hand-curated Motifs. A gene belongs to the regulon if the motif matches the 600 bp upstream region

ChIP-based Regulons
ChIP-chip data Harbison *et al. (2004)*. A gene is belongs to the regulon if the p-value for TF binding is smaller than 0.001

RegulonProfiler
Use t-test to score differential expression of all regulons containing >6 genes; the highest and lowest expression value are discarded

RegulonProfilerDB
(database of regulon profiles)

*regulon*Profiler tutorial ChIP based regulons Motifs based regulons

Experiment	Author	Conditions	PubMed	Supplemental data
C-S-P-N chemostat limitation	Boer	6	PUBMED	data
Cell wall perturbant	Boorsma	2	PUBMED	data
Lithium response	Bro	4	PUBMED	data
Sporulation	Chu	9	PUBMED	data
Carbon-limited chemostats	Daran-Lapujade	6	PUBMED	data
regulation by PDR1	Devaux	11	PUBMED	data
Compounds and stress	Egenomix	20	PUBMED	data
Proteasome inhibitor	Fleming	30	PUBMED	data
Environmental stress	Gasch	180	PUBMED	data
Map kinase	Harris	22	PUBMED	data
Rosetta - gene deletions and compounds	Hughes	300	PUBMED	data
Cell wall mutants	Lagorce	5	PUBMED	data
TCA cycle mutants	McCammon	16	PUBMED	data
Titratable promoter alleles	Mnaimneh	215	PUBMED	data
Cold shift	Sahara	5	PUBMED	data
Cell Cycle	Spellman	77	PUBMED	data
Anearobic N-C-P-S chemostats	Tai	4	PUBMED	data
Calcineurin	Yoshimoto	24	PUBMED	data

Search Experiment name Go

Co-Modulation analysis
Compute Pearson correlation between regulon profiles of two different TFs

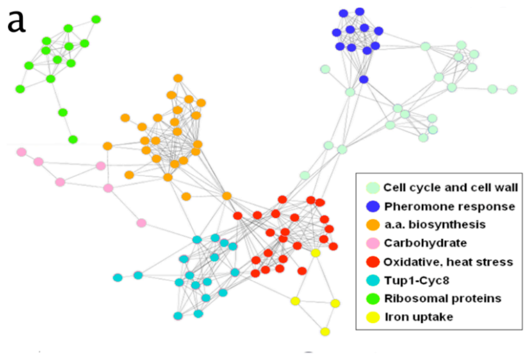

Supplement: Figure S1 — Schematic overview of RegulonProfilerDB. (0.30 MB PDF) [file pone.0003112.s001.pdf]
